# Spinal Implant Osseointegration and the Role of 3D Printing: An Analysis and Review of the Literature

**DOI:** 10.3390/bioengineering9030108

**Published:** 2022-03-06

**Authors:** Cameron Kia, Christopher L. Antonacci, Ian Wellington, Heeren S. Makanji, Sean M. Esmende

**Affiliations:** 1 Department of Orthopaedic Surgery, University of Connecticut, Farmington, CT 06032, USA; antonacci@uchc.edu (C.L.A.); iwellington@uchc.edu (I.W.); 2 Bone and Joint Institute, Hartford Hospital, Hartford, CT 06106, USA; heeren.makanji@hhchealth.org (H.S.M.); sesmende@oahctmd.com (S.M.E.)

**Keywords:** orthopedics, spine, interbody, fusion, 3D printing

## Abstract

The use of interbody implants for spinal fusion has been steadily increasing to avoid the risks of complications and donor site morbidity when using autologous bone. Understanding the pros and cons of various implant designs can assist the surgeon in choosing the ideal interbody for each individual patient. The goal of these interbody cages is to promote a surface area for bony ingrowth while having the biomechanical properties to support the axial skeleton. Currently, the majority of interbody implants consists of metal or polyether ether ketone (PEEK) cages with bone graft incorporated inside. Titanium alloy implants have been commonly used, however, the large difference in modulus of elasticity from bone has inherent issues. PEEK implants have a desirable surface area with the benefit of a modulus of elasticity closer to that of bone. Unfortunately, clinically, these devices have had increased risk of subsidence. More recently, 3D printed implants have come into the market, providing mechanical stability with increased surface design for bony ingrowth. While clinical outcomes studies are limited, early results have demonstrated more reliable and quicker fusion rates using 3D custom interbody devices. In this review, we discuss the biology of osseointegration, the use of surface coated implants, as well as the potential benefits of using 3D printed interbodies.

## 1. Introduction 

Musculoskeletal conditions are among the most disabling and costly conditions experienced by Americans [1]. Spinal fusions have evolved into a viable treatment modality to treat chronic back pain and restore patients’ quality of life [2,3,4]. While autologous bone grafts are generally regarded as the standard augment for spinal fusion surgeries due to their osteogenic capabilities, complications and morbidity to the donor site have given rise to the use of substitutes and spinal implants [5,6]. As the baby-boomer generation continues to age and comprise a disproportionate amount of the musculoskeletal complaints, there is increased need for surgical technologies that utilize the intrinsic regenerative capacity of mineralized tissues to provide more permanent solutions for spinal pathologies [7]. 

Osseointegration refers to the direct integration of bone to metal resulting in structural and functional integration between the living bone and implant surface [8]. Advances in osseointegration have stemmed largely from the dental implant field, which has demonstrated that achieving successful bone implantation requires a strong and direct interaction between bone and implant surface [9,10,11,12,13]. Early literature on osseointegration was problematic, with reports of formation of fibrous capsules around metallic or polymeric implants leading to early failure [14,15,16,17,18,19,20,21]. There have also been reported cases in which significant osteolysis developed, despite indetectable traces of wear debris [21,22,23]. More recently, these issues have been minimized by controlling specific implant properties like surface roughness and nanostructures to promote bone apposition directly onto implant surfaces [24,25,26,27,28,29].

There are a variety of clinically available cages for spinal fusion that differ in shape, texture, and chemical composition. The purpose of this review is to (1) evaluate the key biological processes that occur around implants, (2) discuss the role that surface structure plays on osseointegration, and (3) discuss current literature on custom 3D printed cages and their impact on fusion rates.

## 2. The Biology of Osseointegration

Osseointegration begins with absorption of water molecules, proteins, and lipids to the implant surface [30,31]. The specific host response to the implant surface is dependent upon protein properties [31]. Proteins such as fibronectin and vitronectin tend to initiate inflammation through the attachment of platelets [31,32,33]. These platelet attachments have shown to lead to fibrin clot formation that contributes to a meshwork architecture, which facilitates cell migration toward the implant surface [7,34]. Neutrophils and macrophages utilize this meshwork to rid the area of pathogens and necrotic tissue [35,36]. Mesenchymal stem cells (MSCs) arriving at the implant surface are exposed to these inflammatory cytokines and influence subsequent differentiation into osteoblasts, chondrocytes, and fibroblasts [37,38]. Gittens et al. demonstrated that these MSCs tend to form bone and soft tissue in this environment, although the characteristics of the implant are thought to influence this process [7].

Upon fibrin meshwork formation, bone can form on the bone surface surrounding the implant (a process termed distance osteogenesis) and on the implant surface (contact osteogenesis) [39]. Osteoblasts encountering these two surfaces may reproduce for a few generations or lay down proteins to form the lamina limitans [39]. Often referred to as the cement line [39,40,41], the lamina limitans contains a protein profile which further induces osteoblast migration and maturation. 

**Figure 1:**
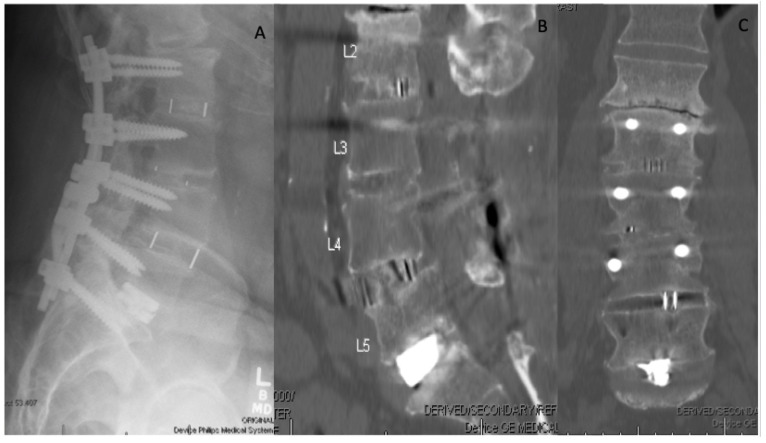
Lateral X-ray of the lumbar spine. (**A**) Demonstrating L2–S1 anterior interbody fusion using a combination of PEEK and titanium cages that is supported posteriorly with pedicle screw fixation. One year post-op CT sagittal (**B**) and coronal slices (**C**) demonstrate bridging callus between the interbody spaces showing successful fusion with osseointegration.

Bony remodeling then occurs, a process in which osteoclasts resorb the newly formed bone to amend microcracks and prepare the surface for new bone formation (Figure 1) [42]. This involves creating resorption lacunae with nanotopography thought to signal osteoblasts that an appropriate surface has been established for new bone formation [7]. If the surface properties are insufficient, migrating cells can form fibrous tissue between implant and bone, resulting in degradation of surrounding bone and implant loosening [24]. 

## 3. Implant Composition

Patient-specific conditions such as old age, poor bone quality, and smoking can threaten the outcome of successful fusion [40]. The goal of implant design is thus to minimize the effects of patient variables and improve implant-bone osseointegration by being cognizant of implant chemical composition, as well as the microscale (1 dimension <100 μm), submicroscale (1 dimension <1 μm), and nanoscale (1 dimension <100 nm) surface characteristics [40]. It is important to acknowledge that the region of the instrumented spine may have relevance for implant selection. Higher reported rates of subsidence and non-union in lumbar fusions compared to other parts of the spine underscores the need for careful implant development and selection when instrumentation of these levels is being planned. 

### 3.1. Titanium and Titanium Alloys

**Figure 2:**
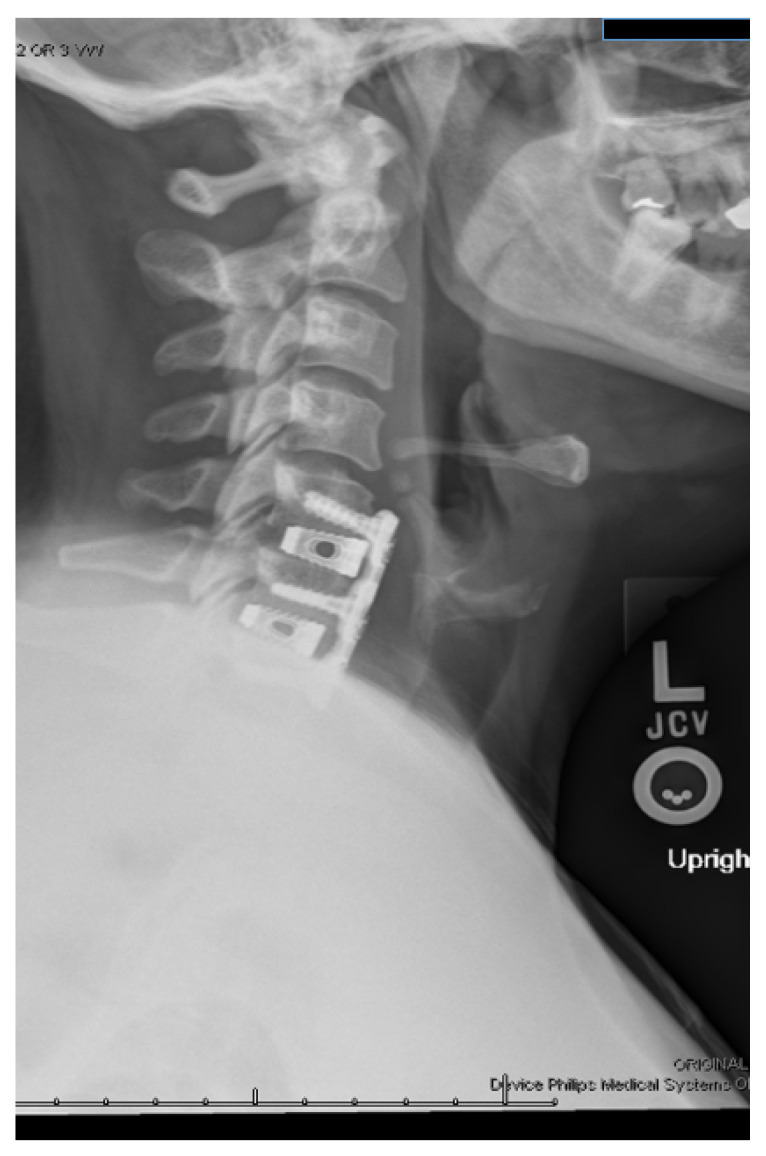
Cervical spine X-ray demonstrating a C5–C7 anterior discectomy and fusion using titanium interbodies with plate fixation.

Titanium and titanium alloy implants are widely used due to their suitable weight-to-strength ratio and good biological performance (Figure 2). Brånemark et al. demonstrated that the surface of titanium allows for osseointegration [9]. Upon exposure to air, titanium forms a thin oxide layer that inhibits further implant corrosion; the titanium oxide film also restricts the release of ionic and molecular titanium species, protecting the biological surroundings from the highly reactive metal [43]. It is suggested that the oxide later provides titanium’s good biological performance by mimicking the ceramic properties of hydroxyapatite in bone [25]. Titanium is relatively agreeable to the spine because of its biocompatibility, robust repassivation, resistance to corrosion, and low density [44]. 

The limitation of titanium alloys revolves around the mismatch in the elastic modulus between titanium (100 GPa) and bone (10–30 GPa) [10]. This mechanical difference can lead to stress shielding around the implant, local inflammation, bone atrophy, subsidence, and implant failure [10]. Moreover, assessing successful fusion radiographically may be difficult due to titanium’s high radiodensity [45]. 

### 3.2. Polyether Ether Ketone (PEEK)

**Figure 3:**
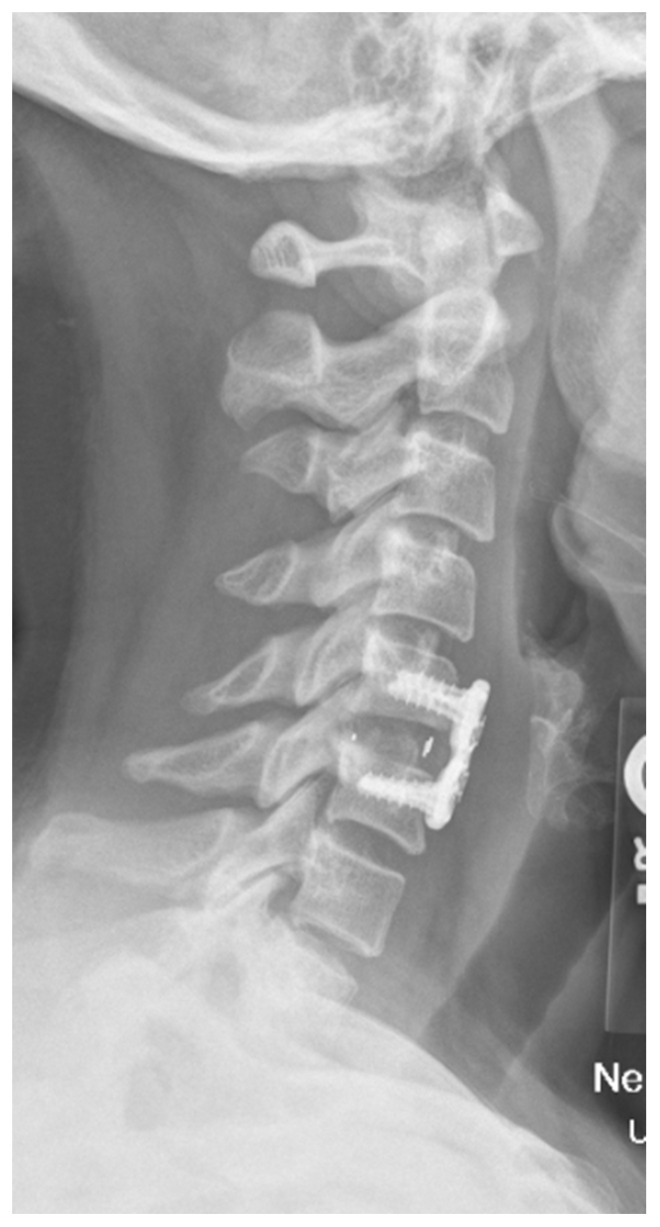
Cervical spine X-ray demonstrating polyether ether ketone (PEEK) interbody after a C5–C6 anterior cervical discectomy and fusion.

Interbody cages are often made of polyether ether ketone (Figure 3), which is an inert semicrystalline polyaromatic linear polymer. PEEK is an inexpensive, radiolucent material that has a modulus of elasticity that approximates that of cortical bone [16]. Its comparable modulus of elasticity to bone has contributed to comparable arthrodesis rates between PEEK cages and autografts [16]. Unlike titanium, the radiolucent composition of these cages allows monitoring of bone growth on post-operative serial radiographs. In contrast to roughened titanium alloy surfaces that promote osteogenesis, PEEK surfaces result in the formation of fibrous tissue [46]. As a result, PEEK cages are often packed with bone graft to achieve vertebral fusion. 

Prospective clinical studies comparing PEEK to autograft have suggested equivalent results in regard to patient-reported outcomes [47]. However, surgical outcomes have suggested higher rates of PEEK cage subsidence, ranging from 32% to 38% of cases [48,49]. Suggested theories for this include over-distraction, overly aggressive endplate preparation, or normal fusion processes [48].

Manipulation of the quantity and direction of fiber element to achieve desired material properties provides PEEK cages with the potential to minimize stress shielding compared to solid titanium implants [50]. Pelletier et al. performed a study comparing PEEK and titanium anterior lumbar interbody fusion implants in sheep, demonstrating no difference in initial biomechanics, mechanical properties, or fusion rates when similar amounts of bone graft were used [50]. There are varying reports in the literature of the interaction between PEEK and osteoblasts [51,52]. Olivares-Navarrete et al. found that osteoblasts differentiate to a lesser phenotypic degree on PEEK versus titanium surfaces, suggesting that PEEK cannot support osteogenic tissue as well as titanium [51]. However, Sagomonyants et al. demonstrated that PEEK and roughened titanium have comparable in vitro bone forming capacity [52]. 

Unlike comparisons between allograft/autograft and PEEK cages suggesting worse subsidence rates with PEEK, comparisons with titanium compounds have had different results [53]. A randomized controlled trial by Chen et al. comparing PEEK to titanium cages in the cervical spine showed subsidence rates of 34.5% in titanium compared to 5.4% in PEEK [53]. Several subsequent studies demonstrated that PEEK preserves intervertebral heights and Cobb angles more effectively than their metallic counterparts [54,55]. 

### 3.3. Surface-Coated Cages

The development of surface-coated interbody cages arose from the abovementioned conflicting evidence regarding PEEK cages and the literature comparing the advantages and disadvantages of PEEK, metals, and biological supplements [53,54,55]. Surface-coated interbody cages increase bone-to-implant contact ratio and bioactivity [53]. Interbody models may be covered with a thin layer of various metals including hydroxyapatite, titanium, gold, titanium dioxide, diamond-like carbon, and tert-butoxides [56]. Hydroxyapatite is the most commonly bioactive material used, with several studies demonstrating increased osteoconductivity of hydroxyapatite-coated PEEK cages, in addition to potentially having osteoinductive properties [56]. 

Surface-coated interbody cages are thought to induce bony overgrowth and arthrodesis starting at the bone–cage interface due to the rough nanometer surface [55]. This surface promotes bony fusion and induces calcium phosphate deposit [55,56,57]. Titanium and gold coating, for example, has been shown to promote osteoblast adhesion on the PEEK interbody graft [58]. Despite these promising results, surface-coated interbody cages have been subject to scrutiny because their modulus of elasticity can range from 10 GPa to 100 GPa (compared to 1.0–2.4 GPa in cortical bone), depending on the density of the coat [53]. Titanium-coated interbody cages have been shown to increase shear strength between implant and bone, reducing the risk of pseudarthrosis, though there is risk of delamination [53]. Kienle et al. performed a biomechanical study to simulate the impaction process in titanium-coated PEEK cages [59]. In contrast to surface-etched implants, the titanium-coated PEEK implants were susceptible to impaction-related wear debris, half of which was of a size range that allows phagocytosis, thus promulgating a systemic inflammatory reaction and possibly hindering arthrodesis [59].

## 4. Surface Modifications

### 4.1. Additive and Subtractive Manufacturing

The two primary types of manufacturing spine implants are additive and subtractive [60]. Additive manufacturing, often termed 3-dimensional (3D) printing, involves the application using computer software or 3D material coating on the implant [61]. Subtractive manufacturing involves generation of surface features through removal of material, producing submicron surface textures; examples of which include acid etching and grit blasting of titanium surfaces, which have been shown to increase osteoblastic differentiation and improve osseointegration and bone formation [62]. 

### 4.2. Microroughness and Nanostructures 

Nanoscale surface modifications represent a developing subfield of fusion science whereby host cells are able to interact with implants on a molecular level via cellular membrane receptors to trigger osteoblastic-lineage [60]. This process occurs on nanoscale (10^−9^ m) surfaces, as microscale (10^−6^ m) surface texture does not interact with cellular membranes [60]. 

Implant surface roughness can be manipulated to influence particular protein families that stimulate certain types of cells to attach. This has been shown to occur with metallic surfaces, PEEK, and hyaluronic acid [52]. In addition, surface roughness can influence initial implant fixation by increasing friction and limiting micromotion [52]. Most commercially available implants contain a surface modification to increase roughness, as this has been demonstrated to have beneficial results with in vitro and in vivo analyses [12,26]. Moreover, surfaces with complex microtopography appear to be even more osteogenic than surfaces with only one type of roughness. Surface modification techniques to increase microroughness include acid etching, sand blasting, heat treatments, and anodic oxidation [7].

Olivares-Navarrete et al. previously evaluated the effects of roughened surfaces on implant types [38]. One study demonstrated that osteoblasts exhibit a more differentiated phenotype when grown on machined or grit-blasted titanium aluminum vanadium alloys than when grown on smoother titanium surfaces [38]. This was supported by Sykaras et al., whom found that the highest levels of osteoclast inhibitors (transforming growth factor beta and osteoprotegerin), angiogenesis factors (fibroblast growth factor 2, vascular endothelial growth factor A, and angiopoietin-1), and bone morphogenetic proteins on roughened titanium surfaces compared to smooth titanium and PEEK [12]. In addition, multiple studies have shown that nanoengineered implants increase stimulation of local growth factors, including bone morphogenetic proteins, vascular endothelial growth, and transforming growth factor beta [63,64].

### 4.3. Bioabsorbable Interbody Cages

The notion of a bioabsorbable interbody fusion is appealing because these implants serve to provide structure before being resorbed over time and replaced by host bone. These structures are intended to recreate the extracellular matrix of bone [64]. However, skepticism regarding the value of bioabsorbable implants and their limited use in clinical practice stem from poor osteoconductivity and low primary stability with development of cracks and foreign body reactions [65]. To improve osteoconductivity, nanosized β-tricalcium phosphate (β-TCP) has been incorporated into polylactide (PLA) cages [66]. Cao et al. developed a bioabsorbable cervical fusion cage from PLA and β-TCP that was shown to have greater biomechanical stability in a sheep model, as compared with tricortical iliac crest grafts and PEEK cages, allowing for resorption over time [66]. A subsequent in vivo study by the same group compared the use of a novel polylactide/nano-sized β-tricalcium phosphate bioabsorbable self-retaining cervical fusion cage (BCFC) to autologous bone graft and PEEK cages [66]. The authors found that at 12 weeks post-operatively the BCFC group yielded a significantly lower range of motion in axial rotation than both the autologous bone graft and PEEK cage group. Histologic evaluation revealed an increased intervertebral bone volume/total volume ratio and better interbody fusion in the BCFC group than in the other groups [66]. However, this was proven in an animal model with no clinical data to date [66].

### 4.4. Hydroxyapatite Coating 

It is well established that bone demonstrates a strong affinity to implants composed of sintered hydroxyapatite (HA) [67]. HA interbody spaces have been used, but the mechanical properties of HA on its own are not well suited to this application because resistance to fatigue failure is very low [67]. However, it is possible to utilize additive techniques to attach HA to titanium. This HA coating technology has also demonstrated increased bone apposition, increased resistance to pull-out forces, and increased extraction torque for HA-coated stainless steel pedicle screws, though this has not been shown for interbody spacers [68].

## 5. 3D Printing 

**Figure 4:**
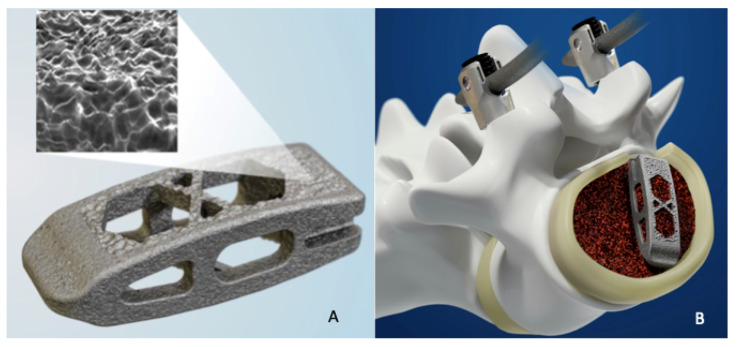
(**A**) 3D printed titanium cage (**B**) with interbody placement. Printed with permission from Medtronic PLC (Minneapolis, MN, USA).

3D printing is a form of additive manufacturing in which multiple 2D layers are formed atop one another to create a 3-dimensional product (Figure 4). 3D printing technology in spine surgery can be used to create models of pathology for pre-operative planning, individualized pedicle screw guides, and most commonly customized implants [69,70]. There are many techniques for 3D printing with the most common being extrusion printing, in which a solid starting material is extruded as either a liquid or semi-liquid and then rapidly cooled. However, orthopedic implants are often printed using powder bed fusion, in which a thin layer of powdered material is deposited on a platform and an electron beam is used to fuse the material to form the implant design [70]. While a predominant amount of the literature on 3D implants utilize titanium, there have been promising reports of PEEK printed implants [71,72].

3D printed implants can be separated into two groups: patient specific (PS) and off-the-shelf (OTS). OTS implants are similar to standard implants but utilize 3D printing technology to impart a customizable porosity and stiffness [73]. Alternatively, PS implants are designed using a patient’s pre-operative CT or MRI scans and allow for superior endplate matching, as well as customized amounts of lordosis and height [70]. OTS implants are desirable as they allow for mass manufacturing and thus decreased costs. Current studies investigating OTS 3D implants have been promising. Mokawem et al. utilized OTS 3D printed TLIF and LLIF titanium interbodies impregnated with silicate-substituted calcium phosphate and found solid fusion on CT at one year in 92 of 93 patients [74]. McGilvray et al. found increased fusion mass for OTS 3D printed titanium alloy cages at 16 weeks compared to PEEK and plasma sprayed PEEK in an ovine model [73].

Several case reports of successful implementation of PS implants in the cervical, thoracic, and lumbar spine for neoplasia, degenerative disease, infection, congenital anomaly, and trauma have been published [72,73,75]. One of the greatest theorized benefits of customized 3D printed implants is increased endplate-cage contact resulting in decreased force point-loading. Mobbs et al created a customized ALIF cage and used finite element analysis to compare pressure loading across the endplate [76]. They found that the custom implant resulted in a more even distribution of force along the endplate compared to a generic ALIF implant [76]. In corpectomy surgery, custom printed cages can be created in concert with planned pedicle screw trajectories to allow for interposition of the posterior construct with the corpectomy cage. 3D printing technology also allows for the creation of multilevel cages made to match a corrected sagittal profile for patients undergoing large corpectomies. 

Custom 3D printed implants have also been successfully used in atlantoaxial fusion. Phan et al successfully used a 3D printed posterior implant with integrated screw holes with a predetermined trajectory for a C1–C2 fusion [77]. While there have been no studies in spine surgery comparing outcomes between OTS and PS implants, there is current literature in knee arthroplasty suggesting PS implants have been associated with less intraoperative blood loss and greater patient reported outcome measures when compared to OTS implants [77]. Further research is warranted to determine if a similar effect is seen in 3D printed spine implants. 

While preliminary reports of 3D printed spinal implants have been promising, this technology is not without its limitations. 3D printed implants, especially PS implants, are more expensive than traditional implants, take longer to produce, and require the technical skills to design and print the final product. As the use of this technology increases, costs are likely to go down, however, it is uncertain if customized implants will gain traction for more common cases with standard patient anatomy. 

## 6. Clinical Implications and Future Perspective

As 3D printing continues to generate more sophisticated interbody shapes and additive potential, the clinical applicability of 3D cages will continue to broaden. This is expected to have significant clinical applicability, as augmenting implants on microscales and nanoscales to improve fusion rates and decrease subsidence are likely to produce better patient outcomes and satisfaction. Positive in vivo results are a promising first step to bringing surface modifications to clinical practice, but long-term clinical studies are needed to ascertain the full clinical implications of these difference surface features on implant performance. Further prospective clinical studies with longer-term outcomes are needed comparing OTS to custom 3D implants.

## 7. Clinical Implications

The purpose of this review was to evaluate the key biological processes that occur around implants, discuss the role that surface structure plays on osseointegration, and discuss current literature on custom 3D printed cages and their impact on fusion rates. This review was deemed necessary due to rapidly evolving methods of augmenting implants and clinical needs to decrease subsidence and generate quicker fusion rates. Cages afford excellent load bearing of the vertebral column and height restoration of the intervertebral space [4]. While they reduce stress on adjacent vertebral bodies, cages do not inherently provide the stimulus for bone remodeling necessary for fusion [4]. Successful spinal fusion utilizing interbody implants is dependent on several characteristics, including surface roughness, material properties, and adequate endplate preparation. Osseointegration relies on a biologic response of osteoblasts, fibroblasts and integrins to allow for new bone to form within the interbody. This review demonstrates PEEK implants offer the benefit of better visualization of fusion on x-ray with less subsidence than titanium implants. The downside of using PEEK has been the lack of roughness for osseointegration. In the future, 3D printed PEEK implants may be able to correct this downside with further clinical studies pending. 

## Data Availability

Not applicable.
